# Antibacterial and in vivo toxicological studies of Bi_2_O_3_/CuO/GO nanocomposite synthesized via cost effective methods

**DOI:** 10.1038/s41598-022-17332-7

**Published:** 2022-08-22

**Authors:** Asifa Qayyum, Zahida Batool, Mahvish Fatima, Saeed Ahmad Buzdar, Hafeez Ullah, Aalia Nazir, Qaiser Jabeen, Sofia Siddique, Rimsha Imran

**Affiliations:** 1 Institute of Physics, The Islamia University of Bahawalpur, Bahawalpur, Pakistan; 2 Department of Physics, Deanship of Educational Services, Qassim University, P.O.Box 6595, Buraydah, 51452 Saudi Arabia; 3 Department of Pharmacology, Faculty of Pharmacy, The Islamia University of Bahawalpur, Bahawalpur, Pakistan; 4 Department of Physics, University of Engineering and Technology Lahore, Lahore, Pakistan

**Keywords:** Graphene, Biophysics, Applied physics, Biological physics

## Abstract

In this research work, Bi_2_O_3_, Bi_2_O_3_/GO and Bi_2_O_3_/CuO/GO nanocomposites have been synthesized via an eco-friendly green synthesis technique, solgel route and co-precipitation method respectively for the assessment of antibacterial activity as well as in vivo toxicity. The XRD patterns confirm the formation of Bi_2_O_3_, Bi_2_O_3_/GO and Bi_2_O_3_/CuO/GO nanocomposites showing monoclinic structures. Crystallite size and lattice strain are calculated by Scherrer equation, Scherrer plot and Willimson Hall plot methods. Average crystallite size measured for Bi_2_O_3_, Bi_2_O_3_/GO and Bi_2_O_3_/CuO/GO nanocomposites by Scherrer equation, Scherrer plot and WH-plot methods are (5.1, 13.9, 11.5)nm, (5.4, 14.2, 11.3)nm and (5.2, 13.5, 12.0)nm respectively. Optical properties such as absorption peaks and band-gap energies are studied by UV–vis spectroscopy. The FTIR peaks at 513 cm^−1^, 553 cm^−1^ and 855 cm^−1^ confirms the successful synthesis of Bi_2_O_3_, Bi_2_O_3_/GO and Bi_2_O_3_/CuO/GO nanocomposites. The antibacterial activity of synthesized Bi_2_O_3_, Bi_2_O_3_/GO and Bi_2_O_3_/CuO/GO nanocomposites is examined against two gram-negative (*Escherichia coli* and pseudomonas) as well as gram-positive bacteria (*Bacillus cereus* and *Staphylococcus aureus*) at dose 25 mg/kg and 40 mg/kg by disk diffusion technique. Zone of inhibition for Bi_2_O_3_, Bi_2_O_3_/GO and Bi_2_O_3_/CuO/GO at dose 40 mg/kg against *E. coli* (gram − ve) are 12 mm, 17 mm and 18 mm respectively and against *Pseudomonas* (gram − ve) are 28 mm, 19 mm and 21 mm respectively. While the zone of inhibition for Bi_2_O_3_/GO and Bi_2_O_3_/CuO/GO at dose 40 mg/kg against *B. cereus* (gram + ve) are 8 mm and 8.5 mm respectively and against *S. aureus* (gram + ve) are 5 mm and 10.5 mm respectively. These amazing results reveal that Bi_2_O_3_, Bi_2_O_3_/GO and Bi_2_O_3_/CuO/GO nanocomposite as a kind of antibacterial content, have enormous potential for biomedical applications. In addition, the in vivo toxicity of synthesized Bi_2_O_3_/CuO/GO nanocomposite is investigated on *Swiss Albino* mice at dose of 20 mg/kg by evaluating immune response, hematology and biochemistry at the time period of 2, 7, 14 and 30 days. No severe damage is observed in mice during whole treatment. The *p* value calculated by statistical analysis of hematological and biochemistry tests is nonsignificant which ensures that synthesized nanocomposites are safe and non-toxic as they do not affect mice significantly. This study proves that Bi_2_O_3_/CuO/GO nanocomposites are biocompatible and can be explored further for different biomedical applications.

## Introduction

The field of nanoscience and nanotechnology has grown tremendously in recent years, and its development in various industries is expanding all the time^1^.Integration of nanotechnology and nanomedicine have given new prospect to medicinal and therapy industries. Nanoparticles are being employed as fluorescence, antibacterial, diagnostic agents and transfectional^2,3^. In recent years, nanomedicine has provided developing technologies for achieving essential goals including precise cancer diagnosis, therapy as well as reducing toxicities^4^.

It is vital to examine the toxicity and clearing of novel materials for potential medicinal uses^5–7^. In the breakdown of materials, clearance mechanism through kidney and liver are critical. Renal clearance may quickly clear NPs less than 5.5 nm^8^, while reticuloendothelial systems can absorb NPs of 10–15 nm range by Ist-pass extract^9,10^. Liver metabolism can partially remove nanoparticles greater than 50 nm^11–14^. Nanocomposites of metal oxides have gained significant interest in current history owing to unique morphological, photocatalytical, optical, physical, thermal, electric, absorption aspects^15,16^.

Bismuth oxide (Bi_2_O_3_) nanoparticles have recently received a lot of attention as a semiconductor material within bismuth-based products due to its simple as well as distinctive properties^17^. Bi_2_O_3_ nanoparticles have several crystalline phases such as monoclinic, triclinic, tetragonal, cubic and BCC as Lopez et al. have identified several crystal phases of Bi_2_O_3_^18,19^. Bismuth (Bi) is a substance with significant atomic number (Z = 83) and photoelectrical absorption co-efficient larger than Pt, I and Au. High X-ray absorbance makes it ideal for use in cancer treatment and as a contrasting agent^20–24^.

GO is a novel material made up of several carbon atoms layers arranged in 2D lattice^25–30^. GO have two unique regions, one with *sp*^2^ hybrid carbon domain while second with various oxygenated groups^31^. It has promising advantages in sustainable power, electrical gadgets, transistor, photovoltaic cells and detectors among others^32^. Chemical and physical characteristics of any material are strengthened when Bi_2_O_3_ is added to it^33^. Owing to literature, number of experts worked on bismuth oxide and graphene oxide composite. Das et al. prepared Bi_2_O_3_/GO nanocomposite sonochemically supported through hydrothermal technique for absorbing the organic dyes^33^. Manavalan et al. prepared Bi_2_O_3_/rGO nanocomposite sonochemically to detect hormone in serum of rat and human^34^.

Cu is an excellent material for antibacterial applications. Copper is now employed as antibiotic, antifungicide as well as antifouling agent^35^. It was discovered that metal surfaces having copper are now the most efficient in lowering bacterial growth after examining a variety of metal surfaces^36,37^. Copper oxide is p-type semiconductor having 1.2 eV bandgap, has received significant interest in low price, processability, wide surface area and renewability^38,39^. CuO nanoparticles are good contender for making antimicrobial medical equipment, bandages and ointments^40^. CuO nanoparticles have been used in variety of biological studies. Booshehri et al. investigate that copper oxide nanoparticles have higher antibacterial property^41^. CuO nanoparticles have been found to have anticancer properties in A549 lung cancer cells^42^. From historical eras copper and copper oxides have been used in numerous biomedical applications such as tissue repair, grocery bags, and dental standards and so on, owing to unique antibacterial and anticancer abilities^43,44^. Nanocomposites are in demand in hopes of improving biological efficiency while also meeting specific requirements. As a result, GO can provide an appropriate platform for functionalizing or hosting CuO nanoparticles. CuO and GO might be a fruitful combination of two material’s properties leading to a revolutionary series of nanocomposites exhibiting unique properties. As a result, we discovered these hybrid composites worth investigating in our search of materials with improved biological activity (anticancer and antibacterial property)^45,46^.

The current work is aimed on developing an easy method for producing Bi_2_O_3_/GO and Bi_2_O_3_/CuO/GO nanocomposites to improve biomedical applications. The produced nanocomposites are analyzed using number of physical techniques including XRD, UV–vis, SEM and FTIR. Our synthesized nanocomposites are theranostic as well as antibacterial agents. Synthesized nanocomposites have many biological applications and mainly utilized as antibacterial agents. They are used for diagnostic and treatment purpose as well as used in hospitals to kill bacteria as antibacterial agents. *Escherichia coli* and Pseudomonas bacteria are used to investigate the antibacterial activity of synthesized nanocomposites. Main objective of this research is to determine the in vivo toxicity studies of Bi_2_O_3_/CuO/GO nanocomposite. Results of Hematological, biochemistry and pathological test are reported here. Research sheds new light on the in vivo toxicity of Bi_2_O_3_/CuO/GO nanocomposite.

## Experimental

### Materials

Graphene oxide (GO), Bismuth nitrate, Bismuth nitrate pentahydrate (99.9% purity), Copper nitrate trihydrate, ammonia (NH_3_), Cetyltrimethylammonium bromide (CTAB) and ethanol (≥ 99.9% purity) were brought from sigma Aldrich. Extract of mentha leaves was used to synthesize bismuth oxide.

### Synthesis of Bi_2_O_3_ nanoparticles

30 g of washed mentha leaves were immersed in 300 ml of distilled water and heated for 2 h at 80 °C to prepare leaf extract for the synthesis of bismuth oxide. Leaf extract was cooled at room temperature before being filtered through Whatman filter paper. 3 g of bismuth nitrate was dissolved in 15 ml of distilled water at 80 °C and also combined with 30 ml of prepared extract at 80 °C and stirred continuously. After 20 h, Bi_2_O_3_ nanoparticles were obtained. Sample was washed with distilled water as well as dried. Resulted product was heated in furnace at 500 °C for 4 h to remove the impurities.

### Synthesis of Bi_2_O_3_/GO nanocomposite

360 mg of graphene oxide was dissolved in 225 ml of distilled water and stirred for 1 h. 5.24 mg of bismuth nitrate was mixed in above solution along with CTAB and stirred for 40 min. NH_3_ was added drop wise to maintain the pH b/w 9–10. Resulted product was washed as well as dried at 95 °C and then heated at 350 °C for 4 h to get final product Bi_2_O_3_/GO nanocomposites.

### Synthesis of Bi_2_O_3_/CuO/GO nanocomposite

0.5 g of GO was dissolved in 40 ml of distilled water and stirred for 1 h. 0.5 g of Bi (NO_3_)_3_·5H_2_O was mixed in 35 ml of ethanol and stirred for 10 min at room temperature. 5 g of copper nitrate trihydrate Cu (NO_3_)_2_·3H_2_O was mixed in 45 ml of ethanol and stirred for 1 h. All the three solutions of GO, Bi (NO_3_)_3_·5H_2_O and Cu (NO_3_)_2_·3H_2_O were mixed and stirred for 1 h to produce homogenous emulsion of black color. CTAB was added followed by the addition of ammonia (NH_3_) solution drop by drop to maintain the pH above 10, as a result of this, precipitation was accomplished. Precipitates were centrifuged after 4 h of stirring at room temperature and then washed with distilled water until pH was 7. Resulting Bi_2_O_3_/CuO/GO nanocomposite was heated overnight in an incubator at 80 °C.

### Experimental protocols statement

The animal studies were performed according to the ARRIVE guidelines. Additionally, All experimental protocols and animal care procedures were according to the guidelines approved by the institutional Research Ethical Committee; i.e. Pharmacy Animal Ethics committee (PAEC), under reference number PAEC/22/71.

## Results and discussions

### X-ray diffraction

**Figure 1:**
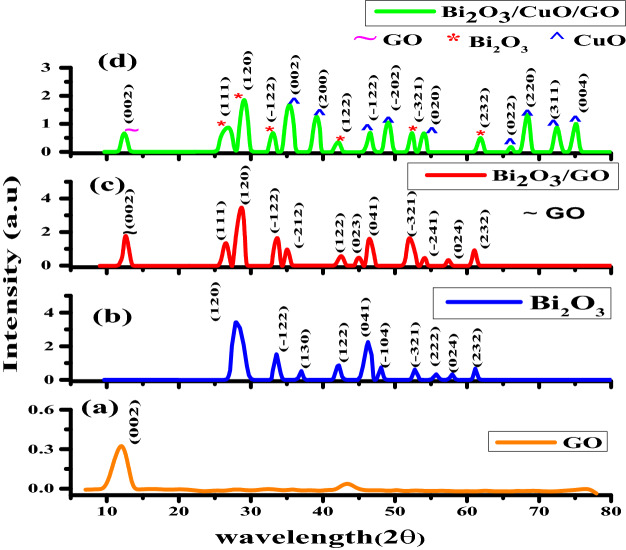
XRD pattern of GO (**a**), Bi_2_O_3_ (**b**), Bi_2_O_3_/GO (**c**) and Bi_2_O_3_/CuO/GO (**d**).

XRD was used to investigate the crystalline structure of Bi_2_O_3_, Bi_2_O_3_/GO and Bi_2_O_3_/CuO/GO nanocomposites. Figure 1a–d demonstrates the XRD patterns of GO, Bi_2_O_3_, Bi_2_O_3_/GO and Bi_2_O_3_/CuO/GO nanocomposites respectively. Cu kα radiations of wavelength (λ) = 1.5406 Å were used in the XRD measurements of prepared NPs. Figure 1b shows the XRD scan of Bi_2_O_3_. XRD peaks of bismuth oxide revealed that nanoparticles have monoclinic structure. All the diffracted peaks with corresponding planes are confirmed by JCPDS card no. 00-041-1449. The sharp peak of bismuth oxide is observed at 27.8° with plane (120). No impurity peak is detected which indicate that the elements reached properly to form bismuth oxide phase. Figure 1c shows the XRD of Bi_2_O_3_/GO composite prepared by sol gel method. Peak at Bragg’s angle 12.5° with plane (002) correspond to the peak of GO^33^. GO peak is wide due to polar–polar interaction between functional groups which contain oxygen in GO^47^. Number of oxygen molecules in the composite increases when Bi_2_O_3_ nanoparticles are added^48^. All the other peaks are in accordance with JCPDS card no. 00-041-1449 of bismuth oxide and high intensity peak occurs at 27.8° correspond to peak of Bi_2_O_3._ Diffraction peaks shows that the structure of nanoparticles is monoclinic. Figure 1d shows XRD of Bi_2_O_3_/GO/CuO nanocomposite. Diffraction peak at angle 12.4° having plane (002) correspond to peak of GO. The diffraction peaks assigned to Bi_2_O_3_ are detected at angle 26.7° (110), 29.0° (120), 33.0° (− 122), 42.0° (122), 52.2° (− 321) and 61.7° (232) have monoclinic phase according to JCPDS card 00-041-1449. The most intense peak of bismuth oxide is at 29°. Most intense diffraction peak at 35.5° with plane (002) is CuO peak according to JCPDS card no. 00-002-1041.All the diffraction peaks assigned to CuO are detected at Bragg’s angle 35.3°, 39.1°, 46.5°, 49.05°, 54.09°, 66.03°, 68.4°, 72.5° and 75.1° with corresponding planes (002), (200), (− 122), (− 202), (020), (022), (220), (311) and (004) respectively having monoclinic phase confirmed by JCPDS card no. 00-002-1041^49^. Lattice parameters, crystal size, interplanar spacing and dislocation density of synthesized materials is shown in Table 1. In ternary nanocomposite Bi_2_O_3_/CuO/GO, the lattice parameters a, b, c and volume is reduced in comparison to binary nanocomposite Bi_2_O_3_/GO. Similarly, the average crystallite size is also reduced 11.5 nm (Bi_2_O_3_/CuO/GO) < 13.9 nm (Bi_2_O_3_/GO) respectively. The decrease in crystallite size increases the surface to volume ratio of the material which is the key factor to various novel properties of material compared to those of the corresponding bulk material. The reduction in average crystallite size is not only important for optical, electronic applications of materials^50^ but also biomedical applications^51^ as well. The average dislocation density of ternary nanocomposite (Bi_2_O_3_/CuO/GO) is decreased which is indication of reduction in grain boundaries which in turn enhance the importance of CuO presence in ternary nanocomposite as compared to binary nanocomposite Bi_2_O_3_/GO.

**Table 1:** Lattice parameters, crystal size, inter planar spacing and dislocation density of synthesized materials.

Sr. no	Material	a (Å)	b (Å)	c (Å)	Volume (m^3^)	Average crystal size (D) nm	Average dislocation density (σ)	Average interplanar spacing (d)
1	Bi_2_O_3_	5.8	8.2	7.5	330.22	5.1	0.0726	0.838
2	Bi_2_O_3_/GO	5.9	8.1	7.51	333.95	13.9	0.014	0.82
3	Bi_2_O_3_/CuO/GO	5.0	5.6	5.4	152.7	11.5	0.009	0.85

#### Scherrer plot method

Scherrer plot methodology was used to investigate the widening of peaks along lattice strain or crystallite size related to dislocation using XRD^52^. Bragg’s peak width is equal to summation of instrumental and sample effect, as calculated by the formula:

$$\begin{aligned} & \beta_{hkl} = \beta_{{{\text{measures}}}}^{2} - \beta_{{{\text{instrumental}}}}^{2} \\ & {\text{D}} = \frac{{{\text{k}}\uplambda }}{\beta cos\theta } \\ & {\text{Or}}\;\,{\text{Cos}}\uptheta = \frac{k\lambda }{D}\left( {\frac{1}{\beta }} \right) \\ \end{aligned}$$

**Figure 2:**
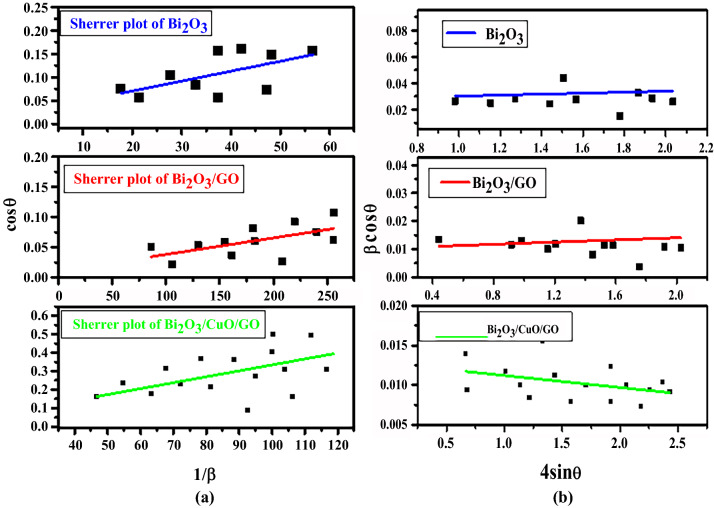
(**a**) Scherrer plot stacking (**b**) WH plot stacking of Bi_2_O_3_, Bi_2_O_3_/GO and Bi_2_O_3_/CuO/GO.

By taking 1/β along x-axis as well as Cosθ along y-axis Scherrer plot was drawn as shown in Fig. 2a. After linear fitting of data, crystal size was estimated from slope of liner line.

#### Williamson Hall (WH) plot method

The W–H analysis is dependent on the assumption that the estimated formulas for strain widening “${\beta }_{s}$” as well as size broadening “${\beta }_{D}$” change in opposite directions when Bragg’s angle (θ) is taken into account. Due to crystal defects and deformation, straininduce widening can occur which is given as.

1 $$\upvarepsilon = \frac{{\beta }_{hkl}}{4 \,tan\theta }$$

Unlike Scherrer plot method, WH method is independent on 1/cosθ but dependent on tanθ. With variation in micro strain and crystal size in the crystal, the difference in 2θ allows us to distinguish b/w strain and size influences on peak widening.

2 $${\beta }_{s} = 4\mathrm{\varepsilon\, tan\theta }$$

By adding strain and crystallite size total peak broadening is attained.

3 $${\beta }_{hkl}= {\beta }_{s}+ {\beta }_{D}$$

Furthermore, the uniform deformation model is used in WH analysis to assume the micro strain to be equal in all crystallographic orientations^52^. ${\beta }_{hkl}$ for this model is

4 $${\beta }_{hkl}= \frac{k\lambda }{Dcos\theta } + 4\mathrm{\varepsilon\, tan\theta }$$

By multiplying cosθ on both sides.

5 $${\beta }_{hkl}cos\theta = \frac{k\lambda }{D} + 4\mathrm{\varepsilon\, tan\theta }$$

Here “ε” is strain. WH plots of are drawn by taking 4sinθ on x-axis and βcosθ on y-axis as shown in Fig. 2b. Micro strain is measured from slope while crystal size is calculated from intercept of linear fitted values^53^. Crystal size by all the three methods WH plot, Scherrer formula and Scherrer plot is shown in Table 2.

**Table 2:** Crystal size by WH plot, Scherrer formula and Scherrer plot.

Sr. no	Samples	Scherrer formula (nm)	WH plot (nm)	Scherrer plot (nm)
1	Bi_2_O_3_	5.1	5.4	5.2
2	Bi_2_O_3_/GO	13.9	14.2	13.5
3	Bi_2_O_3_/CuO/GO	11.5	11.3	12.0

### FTIR analysis

**Figure 3:**
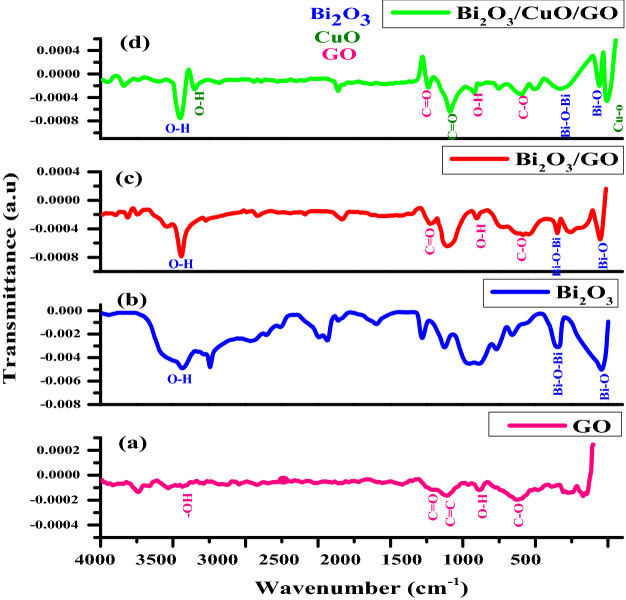
FTIR graphs of (**a**) GO, (**b**) Bi_2_O_3_, (**c**) Bi_2_O_3_/GO and (**d**) Bi_2_O_3_/CuO/GO.

FTIR was used to study functional groups and significance of multiple kinds of functional groups within infrared spectra. FTIR analysis of NPs revealed a number of absorption peaks ranging from 4000 to 400 cm^−1^ as displayed in Fig. 3. FTIR graphs are plotted b/w wavelength (cm^−1^) on x-axis and transmittance (a.u) on y-axis. Figure 3 is the comparison of FTIR graphs of GO (a), Bi_2_O_3_ (b), Bi_2_O_3_/GO (c) and Bi_2_O_3_/CuO/GO. The existence of functional groups such as hydroxyl, epoxide and carboxyl in GO is confirmed by FTIR. In graph (a), the peak observed at 3434 cm^−1^ confirms the existence of hydroxyl group (–OH). At 1723 cm^−1^, the carbonyl group (C=O) is present. At frequency of 1395 cm^−1^, O–H has a bending vibration^54,55^. The presence of epoxide group (C–O) at 1104 cm^−1^ is linked with stretching vibration^56^. The vibration at wavelength 1633 cm^−1^ is due to (C=C) group deposited on GO^57^. In graph (b), metal oxide vibration (Bi–O) is responsible for peak at wavelength 542 cm^−1^ in Bi_2_O_3_. Stretching vibration of O–H is formed at 3430 cm^−1^. In graph (c) two peaks at 543 cm^−1^ and 855 cm^−1^ with corresponding functional groups (Bi–O)^58^ and (Bi–O–Bi) confirms the successful synthesis of Bi_2_O_3_/GO composite. Oxygen containing group (O–H) is present in Bi_2_O_3_/GO composite at 3434 cm^−1^. In graph (d), main peak at 513 cm^−1^ is assigned to Cu–O stretching^59^ while peak at 1601 cm^−1^ is assigned to C=O stretching^60,61^. GO peaks at 1092 cm^−1^,1406 cm^−1^ and 1728 cm^−1^ with corresponding functional groups (C–O), (O–H) and (C=O) respectively as well as peaks at 855 cm^−1^ and 553 cm^−1^ having functional group (Bi–O–Bi) and (Bi–O) respectively are present in Bi_2_O_3_/GO nanocomposite. All these functional groups of GO and bismuth oxide are also present in the composite of Bi_2_O_3_/CuO/GO shown in graph (d). According to characterization the nanocomposites are successfully synthesized.

### UV analysis

#### Optical absorption study

**Figure 4:**
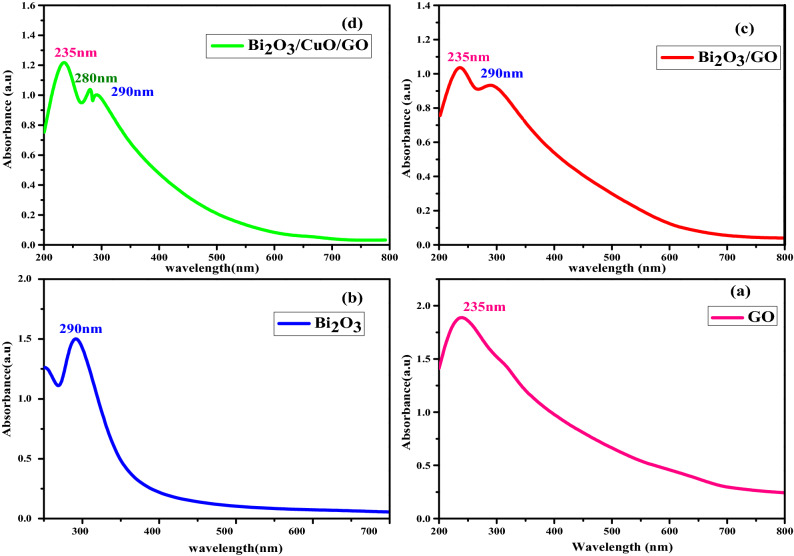
Illustrate the absorption spectra of (**a**) GO, (**b**) Bi_2_O_3_, (**c**) Bi_2_O_3_/GO and (**d**) Bi_2_O_3_/CuO/GO.

Optical absorption studies can be used to evaluate the band structure as well as bandgap energy of semiconductors, non-metallic and metallic materials. Figure 4 illustrates the absorption spectrum of (a) GO, (b) Bi_2_O_3_, (c) Bi_2_O_3_/GO and (d) Bi_2_O_3_/CuO/GO. In pure (a) GO and (b) Bi_2_O_3_, broad absorption peaks are observed at 235 nm and 290 nm respectively^62,63^. In (c) Bi_2_O_3_/GO, composite peak of GO and Bi_2_O_3_ is present which confirms that both oxides exist in single matrix. In graph (d), CuO peak is present at 280 nm as well as GO and bismuth oxide peak is also present which confirms that three oxides exist in one matrix^64^. Single oxide phase scattering caused these absorption peaks in nanocomposite. The extended tail in the absorption spectrum’s wavelength range is most likely caused by scattered radiations from mixed oxide nanoparticles.

#### Bandgap determination

Optical bandgap energy is estimated by this relation:

$$\upalpha {\text{h}}\upupsilon = {\text{A}}\left( {{\text{h}}\upupsilon {-}{\text{E}}_{{\text{g}}} } \right)^{{\text{n}}}$$

A is characteristic factor; υ is frequency of incident light. Bandgap energy is estimated by plotting graph b/w hυ on x-axis and (αhυ)^2^ on y-axis.

**Figure 5:**
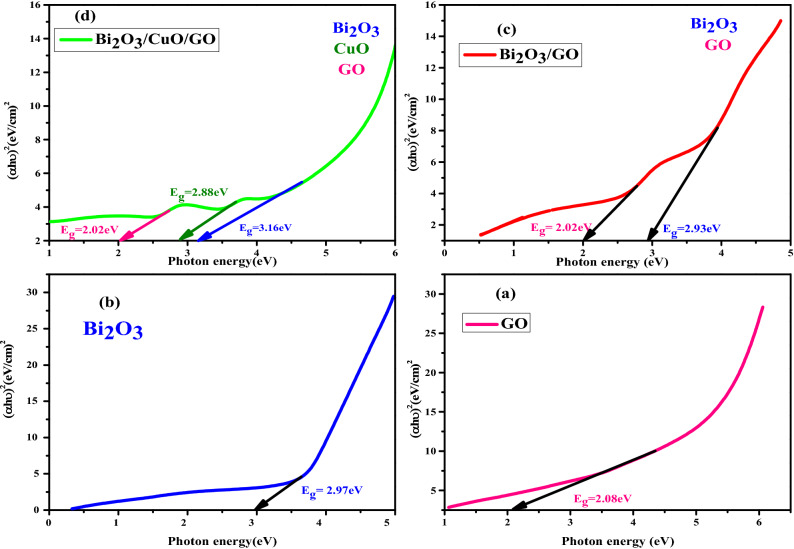
Band gap of (**a**) GO, (**b**) Bi_2_O_3_, (**c**) Bi_2_O_3_/GO and (**d**) Bi_2_O_3_/CuO/GO.

In Fig. 5a and b, bandgap energy value of pure GO and Bi_2_O_3_ is 2.08 eV and 2.97 eV respectively. In graph (c), energy bandgap value of GO is 2.02 eV and Bi_2_O_3_ is 2.93 eV in nanocomposite. In graph (d), value of E_g_ for GO, CuO and Bi_2_O_3_ is 2.02 eV, 2.88 eV and 3.16 eV respectively in Bi_2_O_3_/CuO/GO nanocomposite which demonstrate that composite is composed of GO, Bi_2_O_3_ and CuO^62–64^.

UV analysis revealed that bandgap energy of nanocomposite may be controlled by adjusting volume fraction of the material for a variety of applications including solar cells, solid oxide and photocatalytical activity.

### SEM analysis

**Figure 6:**
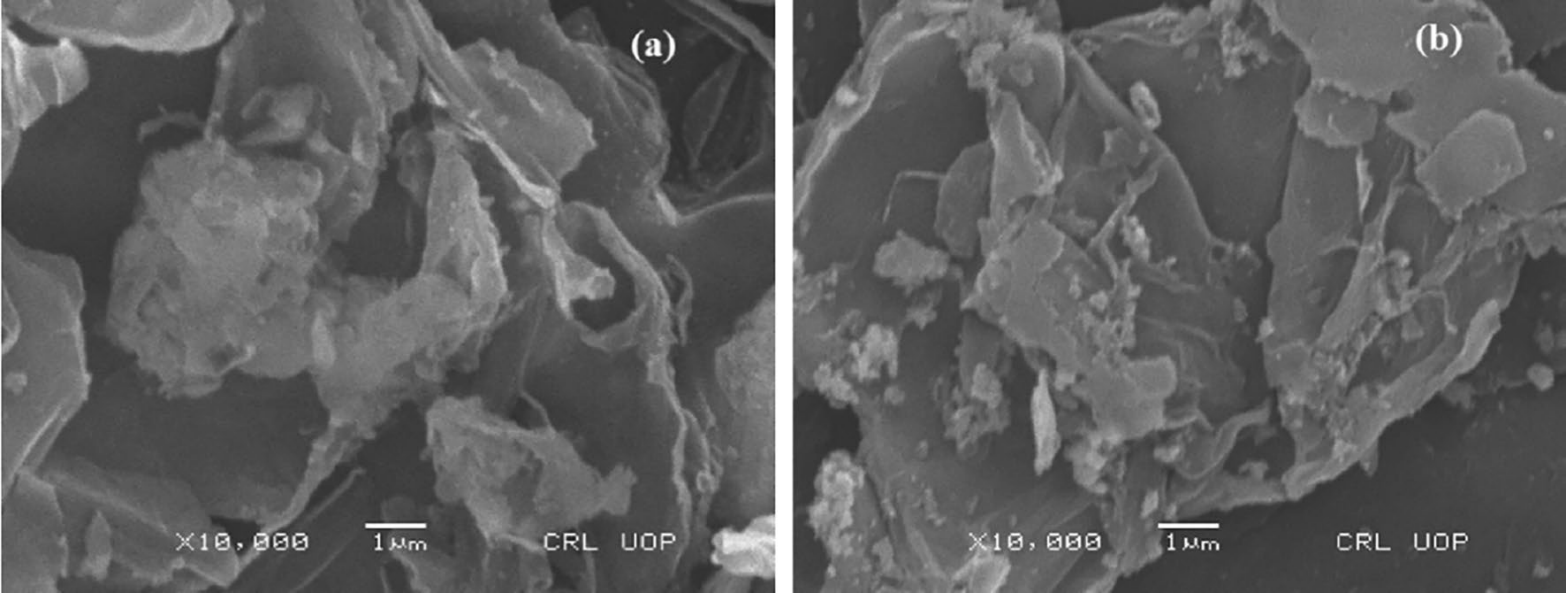
(**a**) SEM image of Bi_2_O_3_/GO and (**b**) SEM image of Bi_2_O_3_/CuO/GO.

The GO structure is sheet like and functional groups which contain oxygen may interact with GO layers and cause folding of GO sheets^65^. In Fig. 6a, GO sheet like structure was observed in nanocomposite containing well-dispersed Bi_2_O_3_ on the surface^66^. The image shows that Bi_2_O_3_/GO nanocomposite surface is rough, may be due to development of NPs of Bi_2_O_3_ on GO sheets^67^. In Fig. 6b, CuO NPs were distributed randomly on GO sheets and data revealed that the sample Bi_2_O_3_/CuO/GO nanocomposites were grouped together and had a rough surface^68^.

### EDX analysis

**Figure 7:**
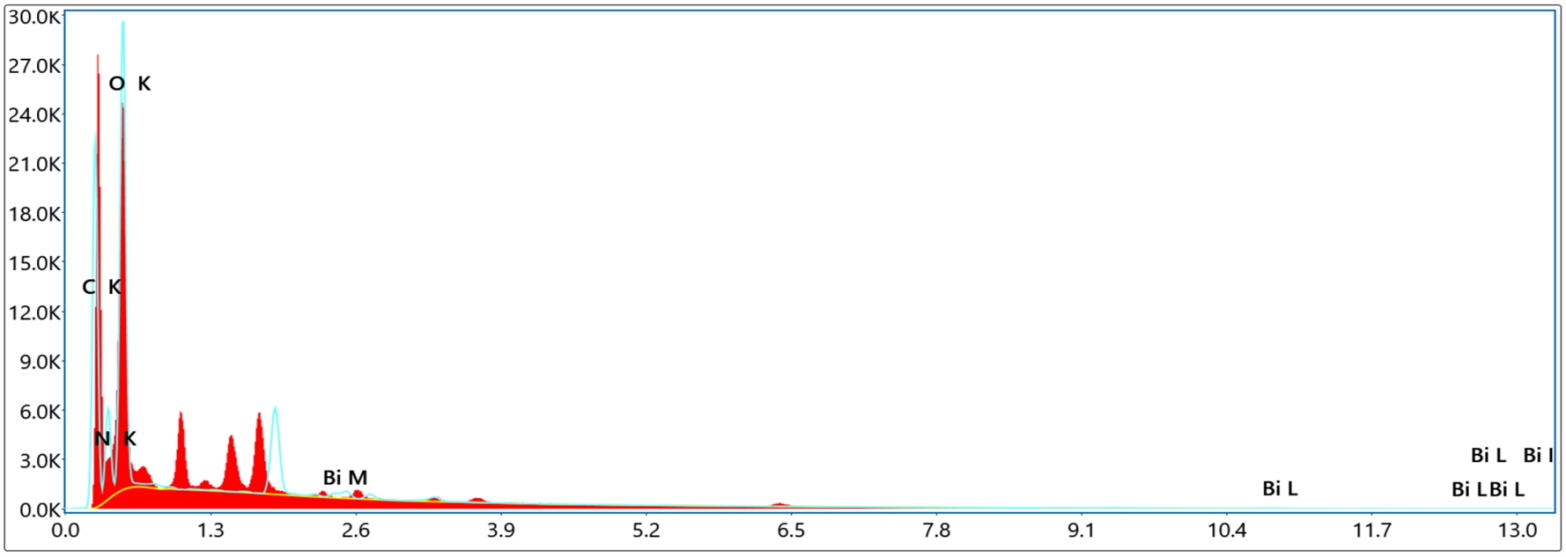
EDX of Bi_2_O_3_/GO.

**Figure 8:**
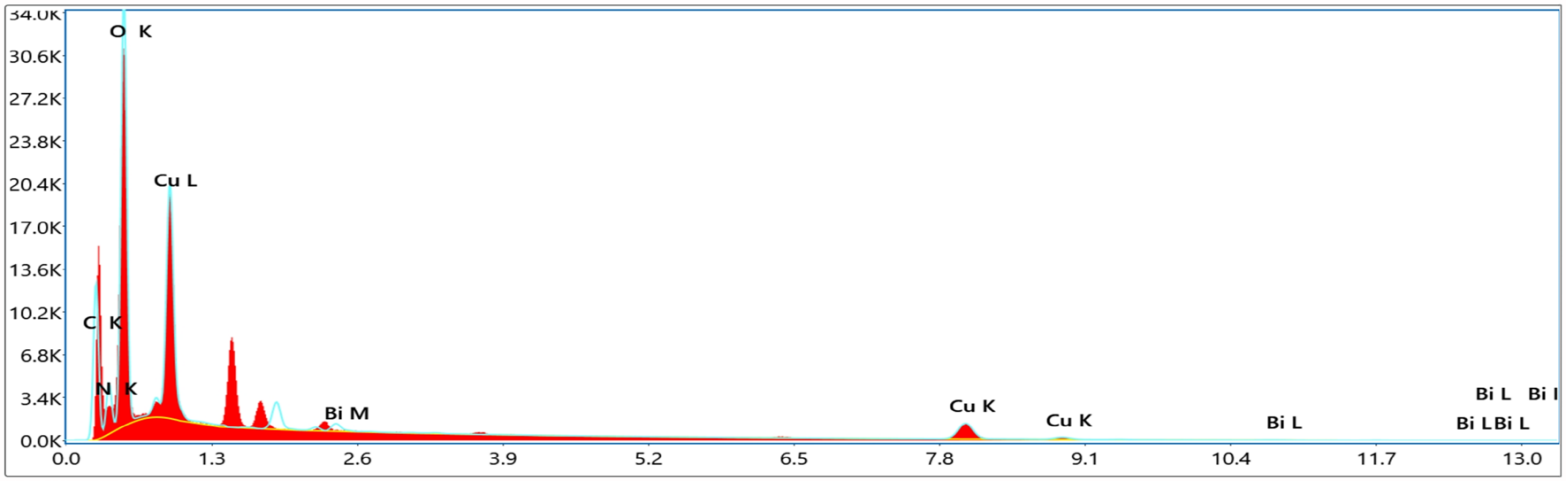
EDX of Bi_2_O_3_/CuO/GO.

Figure 7 shows the EDX of Bi_2_O_3_/GO in which carbon which represents the GO is present. Bismuth is also present in the EDX of Bi_2_O_3_/GO. Similarly, in the EDX of Bi_2_O_3_/CuO/GO bismuth and copper are present with GO which is shown in Fig. 8.

## Antibacterial activity

Antibacterial activity at 40 mg/ml and 25 mg/ml concentration of Bi_2_O_3_, Bi_2_O_3_/GO and Bi_2_O_3_/CuO/GO was performed against *E. coli* and pseudomonad (gram − ve) bacteria as well as *Bacillus cereus* and *Staphylococcus aureus* gram (+ ve) bacteria by disk diffusion technique. First of all, spread bacteria on agar plates then filter papers having synthesized samples were placed on these agar plates. These plates were placed in incubator at 37 °C for 24 h.

### Results of antibacterial activity

**Figure 9:**
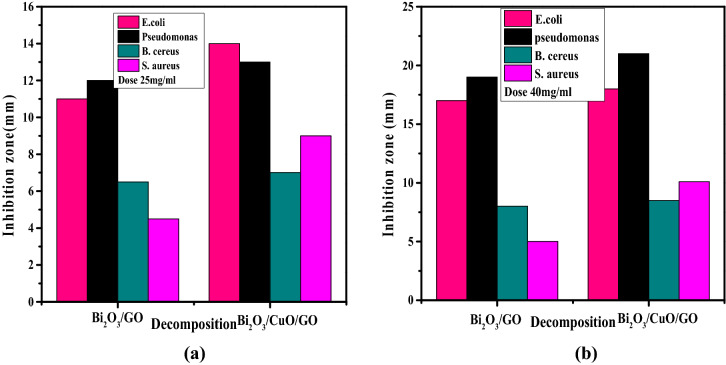
(**a**) The antibacterial activity of synthesized Bi_2_O_3_/GO and Bi_2_O_3_/CuO/GO at dose 25 mg/ml (**b**) the antibacterial activity of synthesized Bi_2_O_3_/GO and Bi_2_O_3_/CuO/GO at dose 40 mg/ml.

**Figure 10:**
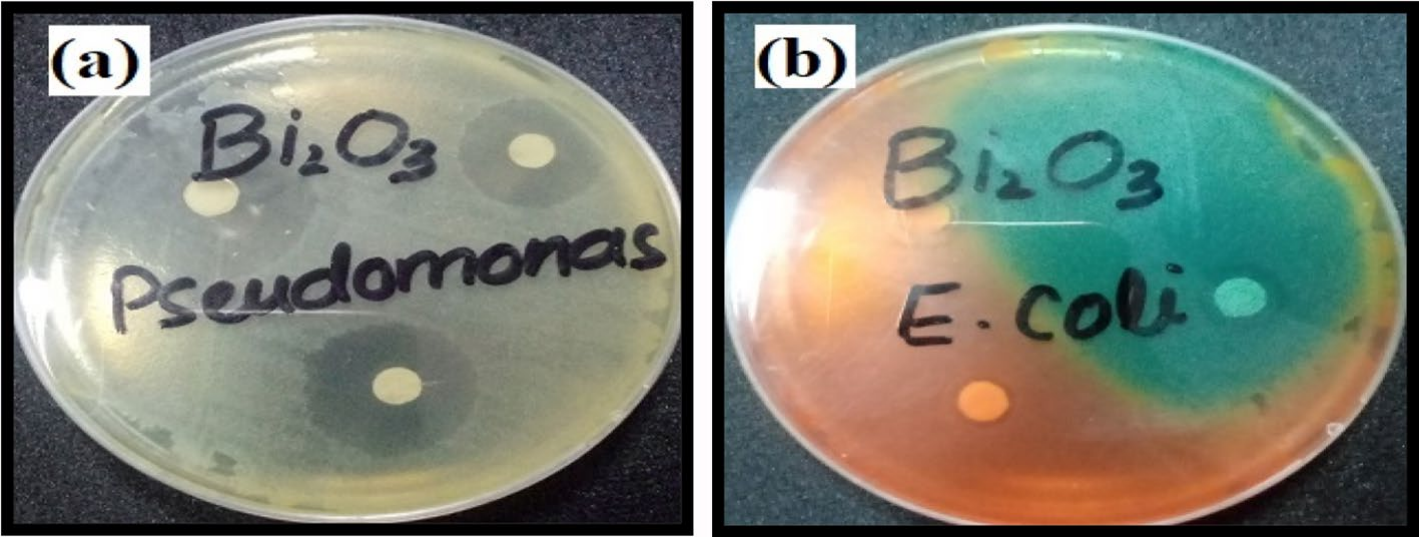
Antibacterial results of Bi_2_O_3_ on (**a**) Pseudomonas gram (− ve) (**b**) *E. coli* gram (− ve) bacteria.

The antibacterial results of prepared Bi_2_O_3_/GO and Bi_2_O_3_/CuO/GO with concentration 40 mg/ml and 25 mg/ml are shown in Fig. 9a and b. Inhibitory zone of pseudomonas and *E. coli* (gram − ve) bacteria measured in mm is comparably higher than *B. cereus* and *S. aureus* (gram + ve) bacteria. Inhibition zone of Bi_2_O_3_/CuO/GO composite is higher than Bi_2_O_3_/GO nanocomposite as shown in Fig. 9a and b. Moreover, the scattering of metal oxides via GO sheets improves the antibacterial property^69^. Figure 10 shows the antibacterial activity of Bi_2_O_3_ against *E. coli* and pseudomonas bacteria. According to Table 3 our prepared samples Bi_2_O_3_, Bi_2_O_3_/GO and Bi_2_O_3_/CuO/GO shown better antibacterial activity than the results mentioned in literature. *Bi*_*2*_*O*_*3*_*/CuO/GO is a novel material which is not reported yet and exhibited enhanced antibacterial effects than other synthesized materials.* Antibacterial activity against gram (+ ve) and gram (− ve) bacteria was performed to compare the results of both gram positive and negative bacteria. Fox 30 and Erythromycins antibiotic were used as reference against gram (− ve) and gram (+ ve) respectively.

**Table 3:** Inhibitory zone of different samples against *E. coli* and pseudomonas.

Sr.no	Sample	Bacteria	Zone of inhibition (mm)	References
1	Bi_2_O_3_	*E. coli*	12	Present
2	Bi_2_O_3_/GO		17	Present
3	Bi_2_O_3_/CuO/GO		18	Present
4	Bi_2_O_3_/GO		6.5	^70^
5	CuO/GO		11.2	^71^
6	CuO		6.6	^61^
7	CHCuO–CH		10	^72^
8	Ag–CuO		17	^73^
9	Bi_2_O_3_	Pseudomonas	28	Present
10	Bi_2_O_3_/GO		19	Present
11	Bi_2_O_3_/CuO/GO		21	Present
12	CuO/GO		14.9	^74^
13	CuO		6.3	^61^
14	Bilayer Wound dressing	*S. aureus*	5.4	^75^
		*E. coli*	1.9	
		*S. epidermidis*	1.0	
15	(PU-HA)1%EEP	*S. aureus*	2.33	^76^
		*E. coli*	1.96	
16	(PU-HA)2%EEP	*S. aureus*	5.63	
		*E. coli*	3.18	
17	PU-WEP	*S. aureus*	3.89	^77^
		*E. coli*	3.55	
18	CS/HA/0.5%EEP	*S. aureus*	2.08	^78^
		*E. coli*	2.64	
		*S. epidermidis*	1.02	

**Figure 11:**
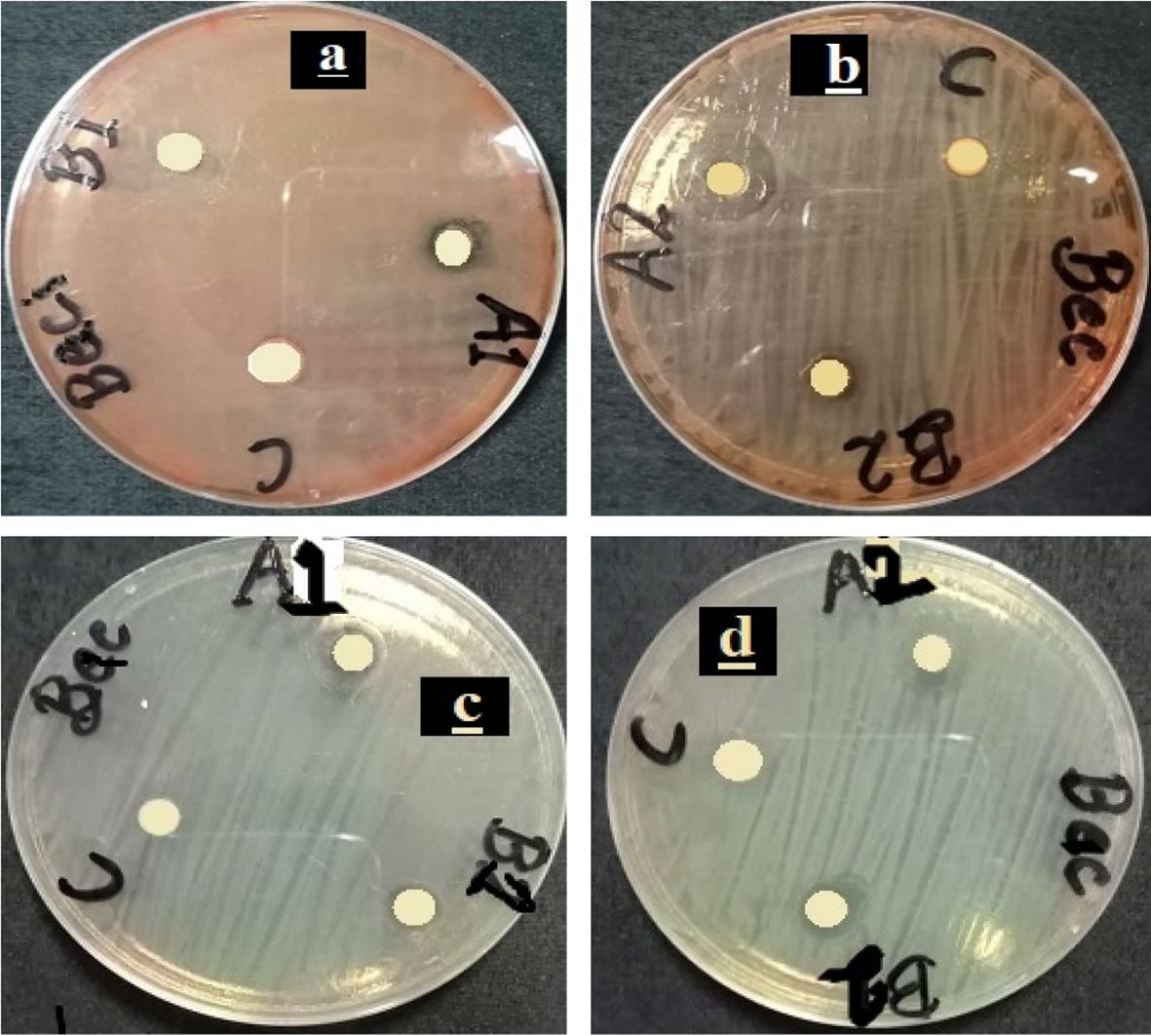
Antibacterial activity of (**a**) Bi_2_O_3_/CuO/GO using dose 25 mg/ml (**b**) Bi_2_O_3_/CuO/GO with dose 40 mg/ml (**c**) Bi_2_O_3_/GO with dose 25 mg/ml and (**d**) Bi_2_O_3_/GO dose 40 mg/ml against *Bacillus cereus* (gram + ve) bacteria.

**Figure 12:**
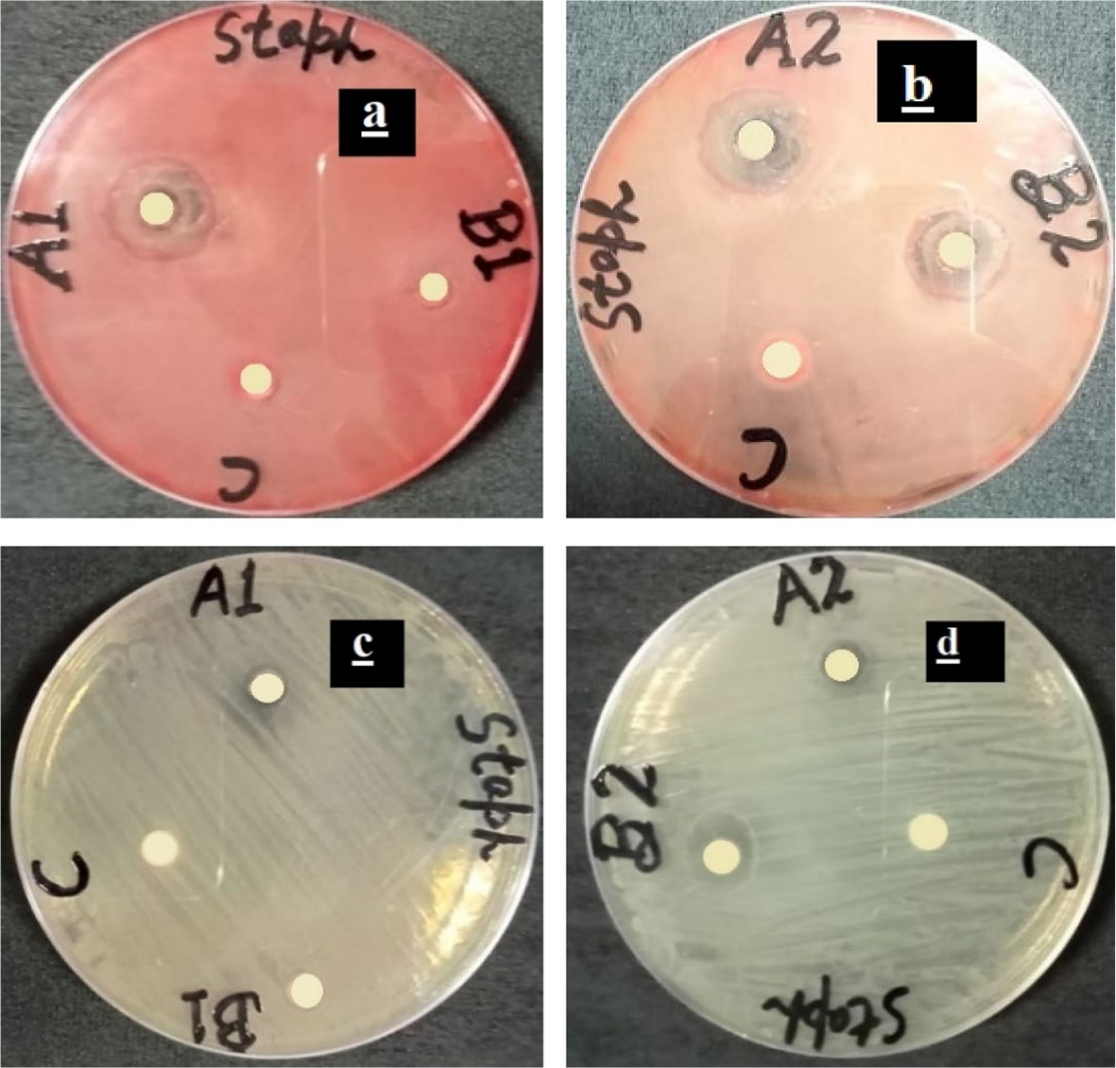
Antibacterial activity of (**a**) Bi_2_O_3_/CuO/GO using dose 25 mg/ml (**b**) Bi_2_O_3_/CuO/GO with dose 40 mg/ml (**c**) Bi_2_O_3_/GO with dose 25 mg/ml and (**d**) Bi_2_O_3_/GO dose 40 mg/ml against *Staphylococcus aureus* (gram + ve) bacteria.

**Figure 13:**
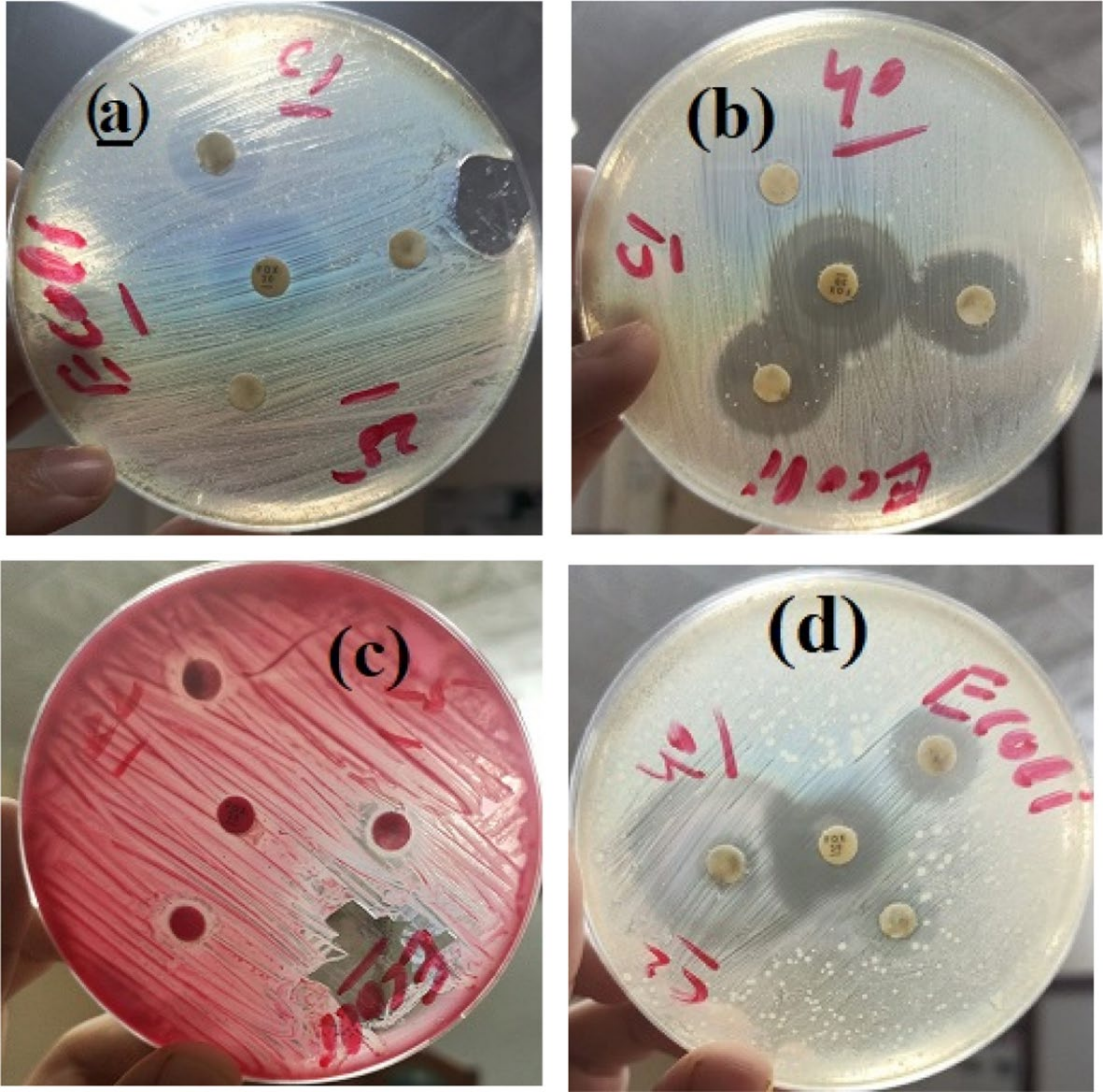
Antibacterial activity of (**a**) Bi_2_O_3_/CuO/GO using dose 25 mg/ml (**b**) Bi_2_O_3_/CuO/GO with dose 40 mg/ml (**c**) Bi_2_O_3_/GO with dose 25 mg/ml and (**d**) Bi_2_O_3_/GO dose 40 mg/ml against *E. coli* gram (− ve) bacteria.

**Figure 14:**
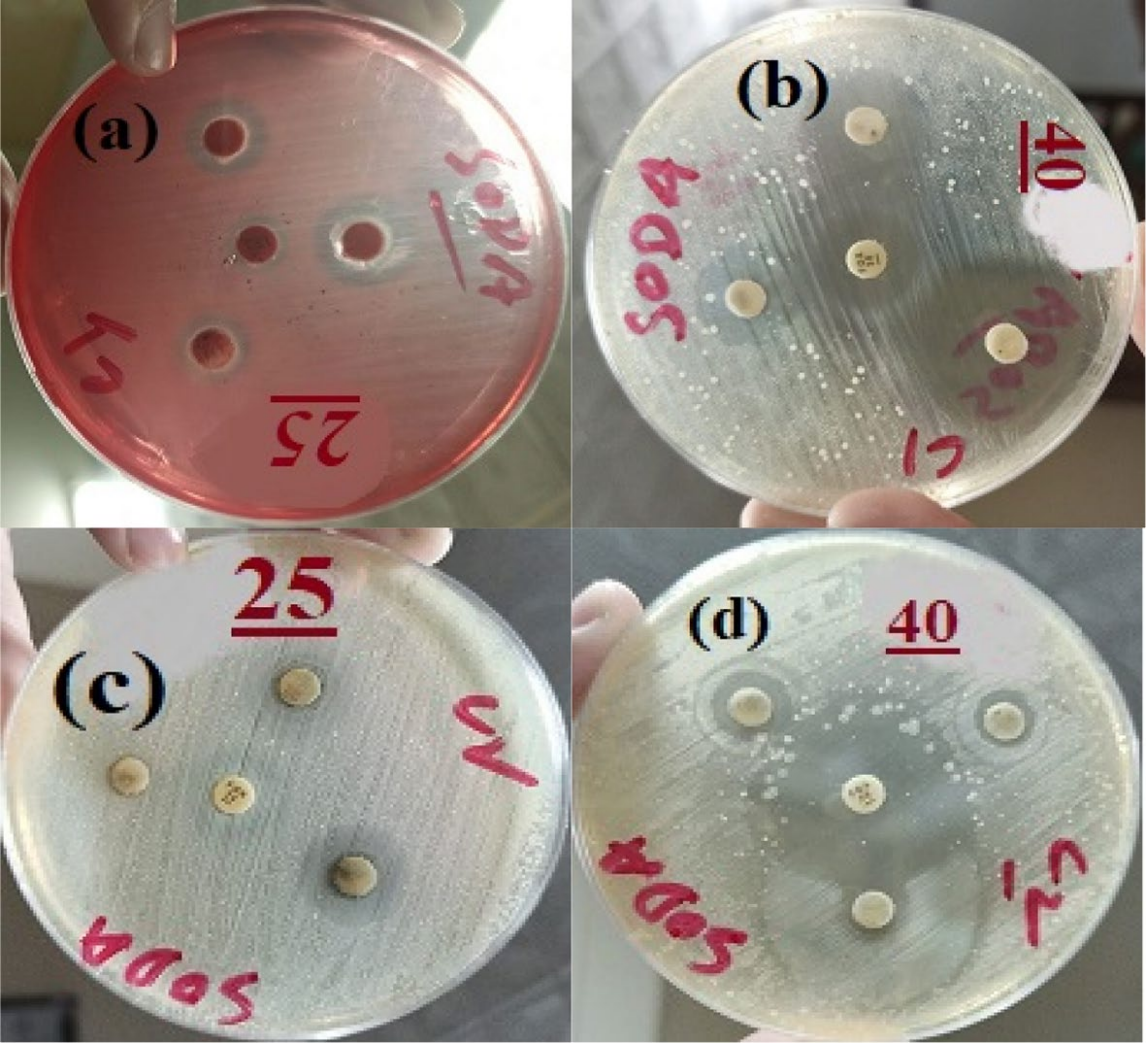
Antibacterial activity of (**a**) Bi_2_O_3_/CuO/GO using dose 25 mg/ml (**b**) Bi_2_O_3_/CuO/GO with dose 40 mg/ml (**c**) Bi_2_O_3_/GO with dose 25 mg/ml and (**d**) Bi_2_O_3_/GO dose 40 mg/ml against pseudomonas gram (− ve) bacteria.

Figure 11 shows *Antibacterial activity of (a)* Bi_2_O_3_/CuO/GO using dose 25 mg/ml (b) Bi_2_O_3_/CuO/GO with dose 40 mg/ml (c) Bi_2_O_3_/GO with dose 25 mg/ml and (d) Bi_2_O_3_/GO dose 40 mg/ml against *Bacillus cereus (gram *+ *ve) bacteria.* Figure 12*shows antibacterial activity of (a)* Bi_2_O_3_/CuO/GO using dose 25 mg/ml (b) Bi_2_O_3_/CuO/GO with dose 40 mg/ml (c) Bi_2_O_3_/GO with dose 25 mg/ml and (d) Bi_2_O_3_/GO dose 40 mg/ml against *Staphylococcus aureus* (gram + ve) bacteria*.* In Figs. 11 and 12 a disc represent the sample; B disc represent the + ve control (Erythromycin) and C shows the − ve control (distilled water). Figure 13 shows the antibacterial activity of (a) Bi_2_O_3_/CuO/GO using dose 25 mg/ml (b) Bi_2_O_3_/CuO/GO with dose 40 mg/ml (c) Bi_2_O_3_/GO with dose 25 mg/ml and (d) Bi_2_O_3_/GO dose 40 mg/ml against *E. coli* gram (− ve) bacteria. Figure 14 shows the antibacterial activity of (a) Bi_2_O_3_/CuO/GO using dose 25 mg/ml (b) Bi_2_O_3_/CuO/GO with dose 40 mg/ml (c) Bi_2_O_3_/GO with dose 25 mg/ml and (d) Bi_2_O_3_/GO dose 40 mg/ml against pseudomonas gram (− ve) bacteria. Results of *E. coli* gram (− ve) and pseudomonas gram (− ve) are better than gram (+ ve) bacteria as shown in Table 4. Almost all the antibiotic discs of Fox 30 shows 18 mm zone of inhibition shown in Figs. 13a–d and 14a and c, while Fig. 14b and c shows zone of inhibition greater than 18 mm.

**Table 4:** Comparison of zone of inhibition against gram (+ ve) and gram (− ve) bacteria.

Sr. no	Sample	Bacteria	Zone of Inhibition (mm) at dose 25 mg/ml	Zone of inhibition (mm) at dose 40 mg/ml	Zone of inhibition (mm) of control
1	Bi_2_O_3_/GO	*E. coli*	11	17	18
2		Pseudomonas	12	19	> 18
3		*B. cereus*	6.5	8	6
4		*S. aureus*	4.5	5	7
5	Bi_2_O_3_/CuO/GO	*E. coli*	14	18	18
6		Pseudomonas	13	21	> 18
7		*B. cereus*	7	8.5	5.5
8		*S. aureus*	9	10.5	8.5

Figures 11 and 12 demonstrate that our sample shows better results than Erythromycin against *B. cereus* and *S. aureus* bacteria.

## In vivo toxicity studies

The in vivo toxicity of Bi_2_O_3_/CuO/GO at 20 mg/kg dose was explored on *Swiss Albino* mice (female) by analyzing body weight, acute toxicity study, hematological as well as biochemistry test at 2, 7, 14 and 30 days. Animals were divided into two groups. One is control group and second is treated group. Each group contain 4 animals. 20 mg/kg dose was administered orally to all the mice of treated group. One mouse from each group was slaughtered at 2, 7, 14 and 30 days. Blood samples and organs (liver, lungs and kidneys) were collected to perform hematological, biochemistry and pathological test. The treatment with Bi_2_O_3_/CuO/GO had no clear deleterious effects on the growth during 30 days period, no immortality was found and there were no significant differences in weight of body b/w Bi_2_O_3_/CuO/GO treated mice and control mice. The acute toxicity parameters such as Alertness, convulsions, grooming, hyperactivity, salivation, lacrimation, sweating, urination, righting reflex, gripping strength, corneal reflex, writhing reflex and pain response were investigated at 30-day time period and no meaningful difference was noticed. The animal studies were performed according to the ARRIVE guidelines. Additionally, all experimental protocols and animal care procedures were according to the guidelines approved by the institutional Research Ethical Committee; i.e., Pharmacy Animal Ethics committee (PAEC), under reference number PAEC/22/71.

### Hematological test

**Figure 15:**
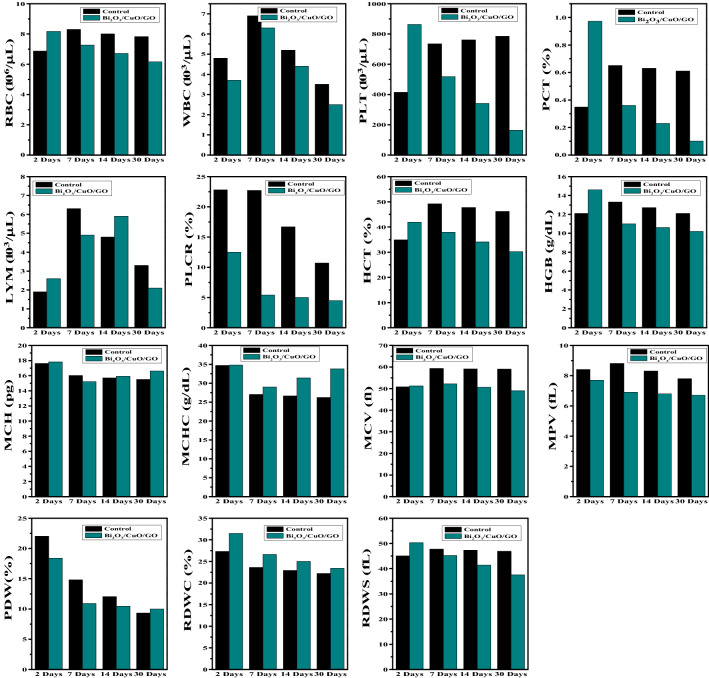
Hematology parameters such as RBC, WBC, PLT, PCT, LYM, PLCR, HCT, HGB, MCH, MCHC, MCV, MPV, PDW, RDWC and RDWS of mice treated with synthesized Bi_2_O_3_/CuO/GO nanocomposite at time period of 2, 7, 14 and 30 days using 20 mg/kg dose.

Hematological parameters such as Hematocrit (HCT), hemoglobin (HGB), lymphocytes (LYM), mean corpuscular hemoglobin (MCH), mean corpuscular hemoglobin concentration (MCHC), mean corpuscular volume (MCV), mean platelet volume (MPV), clotting time (CT), platelet distribution width (PDW), platelet large cell ratio (PLCR), platelets (PLT), red blood cells (RBC), white blood cells (WBC), red cell distribution width—coefficient of variation (RDW-CV) and red cell distribution width—standard deviation (RDW-SD) were examined as shown in Fig. 15.

Two common parameters (WBC and RBC) in Bi_2_O_3_/CuO/GO treated mice were not different significantly from the control mice. Platelets (PLT) and PCT increased after 2 days and then gradually decreases during 30 days treatment. LYM was slightly increases after 2 days and then slightly decreases after 30 days. PLCR was decreases during 30 days of treatment. Hematological parameters such as HCT, HGB, MCH, MCHC, MCV, MPV, PDW, RDWS and RDWC did not change significantly which reveals minor biological impairment.

### Biochemistry test

**Figure 16:**
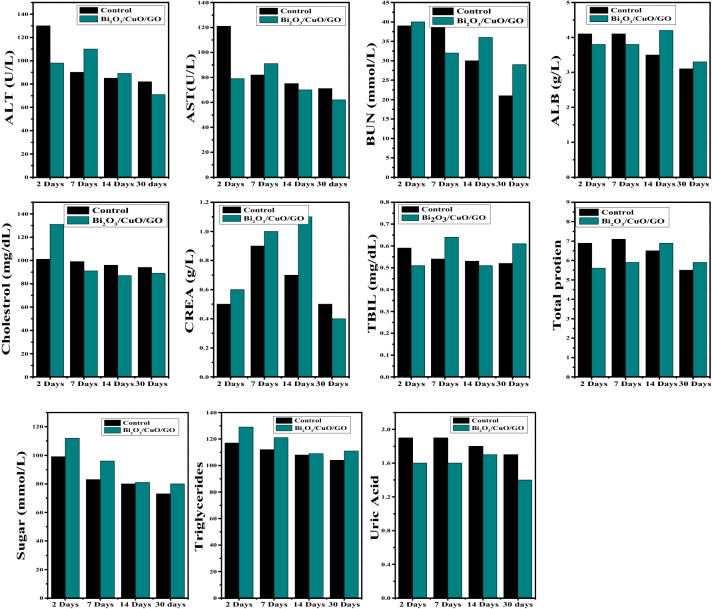
Biochemistry parameters such as ALT, AST, BUN, ALB, Cholesterol, CREA, TBIL, TP, Sugar, Triglyceride and Uric acid of mice treated with synthesized Bi_2_O_3_/CuO/GO nanocomposite at time period of 2, 7, 14 and 30 days using 20 mg/kg dose.

The basic biochemistry tests on mice treated with Bi_2_O_3_/CuO/GO at 2, 7, 14 and 30 days were performed shown in Fig. 16. Biochemistry test such as alanine transaminase (ALT), aspartate amino transferase (AST), albumin (ALB), blood urea nitrogen (BUN), creatinine (CREA), cholesterol, sugar, total bilirubin (TBIL), total protein, triglyceride and uric acid were evaluated. AST, ALT and CREA test were demonstrated as they are strongly linked to liver as well as kidney function of mice. After 2 days of treatment no significant changes were noticed in ALT and CREA but AST was decreased. After 2 days, NPs caused significant liver inflation but did not trigger open wound. No significant change was noticed in ALT and AST after 7 days and 14 days of treatment but CREA was increased after 14 days.

ALT and AST have no significant change after 30 days as well as CREA turned to normal level, indicating that minor damage of liver caused by Bi_2_O_3_/CuO/GO was repaired after 30 days. This is accordance with removal and biodistribution of NPs. The accumulation of oxide-based and Au NPs in liver have been demonstrated to cause a steady increase in ALT and AST as well as substantial liver damage. Au NPs with 5 mg/kg dose level via intravenous administration cause severe injury^79,80^. BUN was slightly increased after 30 days. ALB, cholesterol, sugar, TBIL, total protein, triglycerides and Uric acid were also investigated but no significant change was noticed after 30 days. Fe_3_O_4_ coated with Oleic acid and PEG at dose level 5–7.5 mg/kg caused consistent elevations in ALT, BUN and AST^81^. Even at dose level of 20 mg/kg, Bi_2_O_3_/CuO/GO demonstrates very slight liver damage. These toxicity results are comparable with Bi_2_Se_3_ NPs as reported in literature^82^.

### Pathological tests results

**Figure 17:**
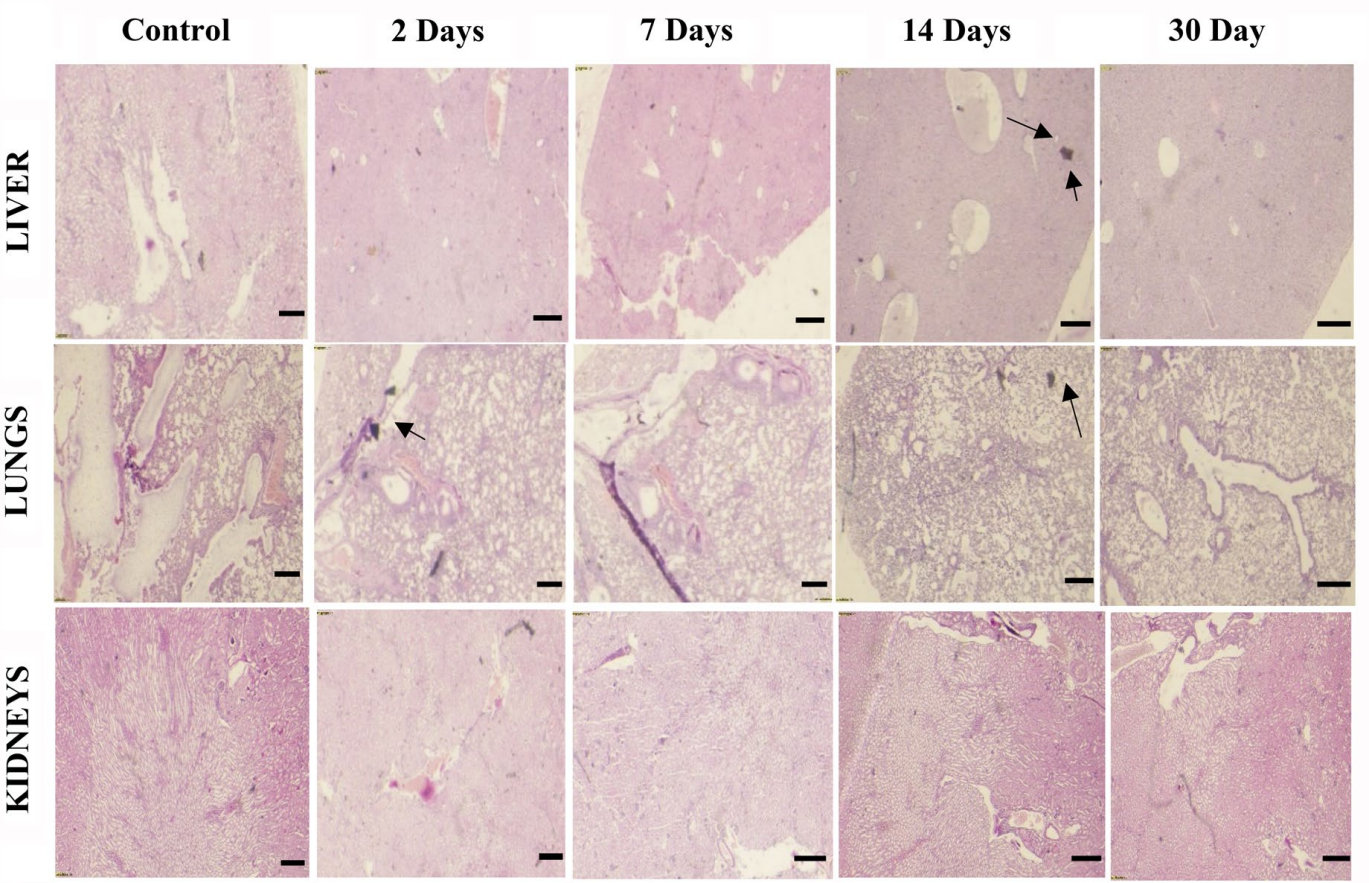
Pathological results of liver, kidney and lungs of mice treated with synthesized Bi_2_O_3_/CuO/GO NPs of dose 20 mg/kg at different time period of 2, 7, 14 and 30 days. Dimensions: 5625.6 µm (W) × 3516 µm (H), scale bar: 87 µm.

We use immunohistochemistry to examine the pathogenic alterations in organs like liver, lungs and kidneys at time period of 2, 7, 14 and 30 days. We collected these organs and prepared the slides of tissues of these organs for microscopy. Throughout the whole treatment session, no damage was identified in kidneys shown in Fig. 17. There was a minor pathological change in liver and lungs was noticed. Very small dark spot was noticed in liver after 4 days but it returned to normal after 30 days. Similarly, a small dark spot was identified in lungs after 2 and 14 days but recovered in 30 days. Additional element of pathology is to analyze the removal of Bi_2_O_3_/CuO/GO NPs qualitatively^81,83^. Whenever NPs assemble in organs, they can accumulate and be recovered directly by using optical microscope. Mice treated with Fe_3_O_4_ NPs, GO and carbon nanotubes had comparable effects.

The liver was found to have black spots, which vanished after roughly 90 days. Now days, Nanomedicine is looking for compounds which have low toxicity and show high clearance efficiency. NPs of small size are commonly believed to be removed through kidneys. But reality on the other hand is significantly more complicated than the assumptions. The clearing of Au NPs is a good example. Au NPs of size 3 nm protected with PEG were not cleared, but Au NPs of same size protected with glutathione were cleared efficiently^79,84^. When the size of carbon materials was between 10 and 30 nm, the kidney and liver may progressively eliminate them^83^. As a result, it is obvious that NPs clearance is influenced not just by size and morphology, but also by shape and durability. Another feasible path for the construction of metabolizable NPs is to investigate the removal of materials of large size. Our current study conclusively demonstrates that Bi_2_O_3_/CuO/GO nanocomposites may be absorbed by liver, indicating that they hold a great potential in medical uses including cancer treatment and contrast agents^82^.

Kefayat et al.^85^ reported the toxicity of albumin stabilized GNPs on BALB/c mice which were injected intra venously with 10 mg/kg dose and sacrificed after 1 month. Through histopathological and biochemistry blood and biochemistry blood analysis as well as histopathological images of organs (liver, lungs, brain, spleen, heart and kidneys) it was observed that GNPs were safe and non-toxic^85^. Toxicity studies of FA-AUNCs on rats with 10 mg/kg dosage injected via intra venous route for a time period of 3 days were reported by Kefayat et al.^86^. By biochemistry analysis and histopathological assays of organs (kidneys, liver and spleen), they concluded that no severe damage was found^86^. Ghahremani et al.^87^, reported that APT-GNCs exhibit no toxic effect on BALB/c mice in a duration of 20 days when a dose of 8 mg/kg was injected intravenously to them.

### Statistical analysis

Tables 5 and 6 shows the *p* value of hematological and biochemistry parameters respectively. GraphPad (2D scientific Graphing) software (version 8) was used to calculate *p* value. From *p* value it is very clear that synthesized nanoparticles have no significant effect on mice and our nanoparticles are safe. *p* Value shows non-significant results. Similarly, Table 7 shows acute toxicity study of mice at 0 h, 2 h, 6 h, 24 h and 48 h and there is no significant change occurs in behavior of mice.

**Table 5:** Hematological parameters of mice treated with synthesized Bi_2_O_3_/CuO/GO nanocomposite.

Parameter	Control group (Mean ± SEM)	Treatment group (Bi_2_O_3_/CuO/GO nanocomposite) (Mean ± SEM)	*p* value
Hematocrit (HCT)	44.50 ± 6.51	36.01 ± 5.02	0.085
Hemoglobin (HGB)	12.55 ± 0.29	11.55 ± 0.96	0.358
Lymphocytes (LYM)	4.07 ± 0.95	3.87 ± 0.91	0.884
Mean corpuscular hemoglobin (MCH)	16.20 ± 0.48	16.63 ± 0.78	0.658
Mean corpuscular hemoglobin concentration (MCHC)	28.63 ± 2.03	32.25 ± 1.29	0.183
Mean corpuscular volume (MCV)	57.06 ± 2.08	50.75 ± 0.67	0.028*
Mean platelet volume (MPV)	8.32 ± 0.20	7.02 ± 0.22	0.005**
Clotting time (CT)	0.56 ± 0.07	0.42 ± 0.19	0.511
Platelet distribution width (PDW)	14.54 ± 2.72	12.44 ± 1.99	0.557
Platelet large cell ratio (PLCR)	18.23 ± 2.88	6.86 ± 1.89	0.016*
Platelets (PLT)	673.80 ± 87.18	471.50 ± 149.40	0.286
Red blood cells (RBC)	7.75 ± 0.31	7.08 ± 0.43	0.251
White blood cells (WBC)	5.10 ± 0.70	4.22 ± 0.79	0.440
Red cell distribution width- coefficient of variation (RDW-CV)	24.00 ± 1.13	26.63 ± 1.75	0.258
Red cell distribution width—standard deviation (RDW-SD)	46.73 ± 0.60	43.59 ± 2.73	0.305

The values are expressed as Mean ± SEM and statistically analyzed using t-test. The results of the treatment group are compared with those of the control group and considered non-significant if *p* > 0.05, significant if *p* < 0.05 and more significant if *p* < 0.01^85^.

**Table 6:** Biochemical parameters of mice treated with synthesized Bi2O3/CuO/GO nanocomposite.

Parameter	Control group (Mean ± SEM)	Treatment group (Bi_2_O_3_/CuO/GO nanocomposite) (Mean ± SEM)	*p* value
Alanine transaminase (ALT)	96.75 ± 11.21	92.00 ± 8.21	0.744
Aspartate aminotransferase (AST)	87.25 ± 11.48	75.50 ± 6.22	0.403
Albumin (ALB)	3.70 ± 0.24	3.77 ± 0.18	0.815
Blood urea nitrogen (BUN)	32.25 ± 4.30	34.25 ± 2.39	0.699
Creatinine (CREA)	0.65 ± 0.09	0.77 ± 0.16	0.537
Cholesterol	97.50 ± 1.55	99.50 ± 10.53	0.857
Sugar	83.75 ± 5.49	92.25 ± 7.53	0.397
Total bilirubin (TBIL)	0.56 ± 0.02	0.57 ± 0.03	0.956
Total protein	6.50 ± 0.35	6.07 ± 0.28	0.386
Triglyceride	110.30 ± 2.78	117.50 ± 4.64	0.229
Uric acid	1.82 ± 0.05	1.57 ± 0.63	0.019

The values are expressed as Mean ± SEM and statistically analyzed using t-test. The results of the treatment group are compared with those of the control group and considered non-significant if *p* > 0.

**Table 7:** The behavioral pattern of mice treated with synthesized Bi2O3/CuO/GO nanocomposite during acute toxicity study.

Parameters	0 h	2 h	6 h	24 h	48 h
Alertness	✓	✓	✓	✓	✓
Grooming	✓	✓	✓	✓	✓
Convulsions	×	×	×	×	×
Hyperactivity	×	×	×	×	✓
Lacrimation	×	×	×	×	×
Salivation	×	×	×	×	×
Urination	×	×	×	×	×
Touch Response	✓	✓	✓	✓	✓
Pain response	✓	✓	✓	✓	✓
Writhing reflex	×	×	×	×	×
Corneal reflex	✓	✓	✓	✓	
Gripping strength	✓	✓	✓	✓	✓
Righting reflex	✓	✓	✓	✓	✓
Skin color	×	×	×	×	×

(✓) = Present.

(×) = Not present/no change.

Table 8 shows the in vivo toxicity comparison of synthesized Bi_2_O_3_/CuO/GO nanocomposites with other reported CuO and GO based composites.

**Table 8:** Toxicity comparison of our synthesized nanocomposites with CuO ad GO based nanocomposites.

Type	Materials	Animal	Mechanism of exposure	Findings	Refs
In vivo	Bi_2_O_3_/CuO/GO	Swiss albino mice	20 mg/kg dose was administered orally for 30 days	Pathology shows small black spots in liver and lungs which disappear after 30 days. According to Hematological and biochemistry results there is no significant damage is found and particles are not toxic	Present
In vivo	rGO/Ag NC	Mice	10 mg/kg dose was injected intraperitoneally for 7 days	According to findings, ALT, AST and creatinine increased implying a negative impact of rGO/Ag nanocomposite on liver and kidneys. Which confirms the toxic effect of green synthesized rGO/AgNC	^88^
In vivo	Cu NPs	Male wister rats	50, 100 and 200 mg/kg dose administered orally for 5 days	Pathological results show that toxicity was induced in both liver and kidneys. In liver, necrosis of tissues and in kidney necrosis in proximal renal tubule as well a swelling of proximal tubule was observed	^89^
In vivo	CuO NPs	Male wister rats	10, 100 and 300 mg/kg dose delivered through IP injection for 14 days	Toxicity was induced in lungs and liver with all concentration of CuO NPs. In liver, vasculature in central veins, portal triad vessels and loss of hexagonal lobules was observed. And in lungs thickening of air scars can be seen	^90^

## Conclusions

Bi_2_O_3_, Bi_2_O_3_/GO and Bi_2_O_3_/CuO/GO nanocomposites were successfully synthesized via green method, sol–gel technique and co-precipitation method respectively. The results of characterization techniques such as X-ray diffraction, Fourier transform infrared spectroscopy, scanning electron microscopy and UV–vis spectroscopy revealed that nanocomposites were successfully synthesized. The crystal size of Bi_2_O_3_, Bi_2_O_3_/GO and Bi_2_O_3_/CuO/GO nanomaterials was 5.1 nm, 13.9 nm and 11.5 nm respectively by Scherrer formula. XRD pattern confirms the monoclinic structure of all synthesized nanomaterials. Antibacterial activity demonstrates that the inhibition zone for Bi_2_O_3_, Bi_2_O_3_/GO and Bi_2_O_3_/CuO/GO nanocomposites against gram − ve *E. coli* is 12 mm, 17 mm and 18 mm respectively and against pseudomonas is 28 mm, 19 mm and 21 mm respectively. In vivo toxicity on *Swiss albino* mice was investigated at dose of 20 mg/kg of Bi_2_O_3_/CuO/GO nanocomposite at different time period of 2, 7, 14 and 30 days. Hematological, biochemistry and pathological results revealed that nanocomposites are less toxic and after 30 days of treatment the slight effects on liver were recovered.

## Data Availability

All relevant data are included in the article.
